# Creating a Multisite Perinatal Psychiatry Databank: Purpose and Development

**DOI:** 10.3390/ijerph17249352

**Published:** 2020-12-14

**Authors:** Wid Kattan, Laura Avigan, Barbara Hayton, Jennifer L. Barkin, Martin St-André, Tuong-Vi Nguyen, Hannah Schwartz, Marie-Josée Poulin, Irena Stikarovska, Rahel Wolde-Giorghis, Maria Arafah, Phyllis Zelkowitz

**Affiliations:** 1 Division of Psychiatry, Department of Medicine, College of Medicine, King Abdulaziz University, Jeddah 21589, Saudi Arabia; wid.kattan@gmail.com; 2 Department of Psychiatry, McGill University, Jewish General Hospital, Montréal, QC H3T 1E2, Canada; aviganl@post.bgu.ac.il; 3 Perinatal Mental Health Service, Department of Psychiatry, McGill University, Jewish General Hospital, Montréal, QC H3T 1E2, Canada; barbara.hayton@mcgill.ca; 4 Community Medicine and Obstetrics and Gynecology, Department of Community Medicine, School of Medicine, Mercer University, Macon, GA 31207, USA; barkin_jl@mercer.edu; 5 Perinatal and Early Childhood Psychiatry Clinic, Sainte-Justine University Hospital Center and Université de Montréal, Montréal, QC H3T 1C5, Canada; martin.st-andre.hsj@ssss.gouv.qc.ca; 6 Department of Psychiatry, McGill University, Montréal, QC H4A 3J1, Canada; tuong.v.nguyen@mcgill.ca (T.-V.N.); phyllis.zelkowitz@mcgill.ca (P.Z.); 7 Department Obstetrics and Gynecology, McGill University, Montréal, QC H4A 3J1, Canada; 8 Ludmer Center for Neuroinformatics and Mental Health Reproductive Psychiatry Clinic, Research Institute of the McGill University Health Center (RI-MUHC), MUHC, Montréal, QC H3T 1J4, Canada; 9 Department of Psychiatry, St. Mary’s Hospital Center, McGill University, Montréal, QC H3T 1M5, Canada; Hannah.schwartz@mcgill.ca; 10 Institut Universitaire en Santé Mentale de Québec, Quebec, QC G1J 2G3, Canada; marie-josee.poulin2.ciussscn@ssss.gouv.qc.ca; 11 Centre intégré universitaire en santé et services sociaux de la capitale nationale (CIUSSSCN), Quebec, QC G1C 3S2, Canada; 12 DHU Ste-Justine, University of Montréal, Montréal, QC H3T 1J4, Canada; irena.stikarovska@umontreal.ca; 13 Department of Psychiatry, University of Montréal, Montreal, QC H3T 1J4, Canada; rahel.giorghis.cnmtl@ssss.gouv.qc.ca; 14 Department of Pathology, King Saud University, Riyadh 11451, Saudi Arabia; 15 Lady Davis Institute for Medical Research, Jewish General Hospital, Montréal, QC H3T 1E2, Canada

**Keywords:** databank, mental health, perinatal health, research

## Abstract

Mental health issues during the perinatal period are common; up to 29% of pregnant and 15% of postpartum women meet psychiatric diagnostic criteria. Despite its ubiquity, little is known about the longitudinal trajectories of perinatal psychiatric illness. This paper describes a collaboration among six perinatal mental health services in Quebec, Canada, to create an electronic databank that captures longitudinal patient data over the course of the perinatal period. The collaborating sites met to identify research interests and to select a standardized set of variables to be collected during clinical appointments. Procedures were implemented for creating a databank that serves both research and clinical purposes. The resulting databank allows pregnant and postpartum patients to complete self-report questionnaires on medical and psychosocial variables during their intake appointment in conjunction with their clinicians who fill in relevant medical information. All participants are followed until 6 months postpartum. The databank represents an opportunity to examine illness trajectories and to study rare mental disorders and the relationship between biological and psychosocial variables.

## 1. Introduction

Perinatal mental health problems are highly prevalent; up to 29% of pregnant women and 15% of postpartum women have symptoms severe enough to warrant a psychiatric diagnosis [1]. These problems can affect women’s wellbeing, mental health and functioning [2,3] and the development of their children [4,5]. Many disorders have a higher prevalence during the pregnancy and postpartum period. For instance, the rate of obsessive-compulsive disorder (OCD) is increased from 1.08% in the general population to 2.07% in the pregnant and 2.43% in the postpartum populations [6]. For other disorders, pregnancy can be a tumultuous period of relapse, such as for women with histories of bipolar affective disorder who have a 50% risk of symptom relapse during pregnancy [7].

Research has shown that maternal mental health issues during the perinatal period not only affect the mother’s wellbeing and functioning, but also have far reaching consequences for obstetric and neonatal outcomes, as well as long-term effects on child development [4,8,9,10].

Greater awareness of these disorders has led to the rise of reproductive psychiatry as an important subspecialty in psychiatry. However, as this is still a relatively recent field of research, there are still few epidemiologic studies of psychiatric episodes related to the perinatal period [11]. The focus to date has largely been on identifying risk factors, screening and symptom assessment in the community [12,13,14].

The focus on community research has led to the conflation of two different populations: those who have perinatal “distress” and those who are diagnosed with major mental “disorders” by clinicians. Murray and Carothers found that many distressed individuals who scored above the threshold on the Edinburgh Postnatal Depression Scale (EPDS) did not actually meet full diagnostic criteria for a major depressive disorder (MDD) [15]. Furthermore, Wisner et al. reported that 2/3 of those screening positive on the EPDS had a comorbid anxiety disorder, and 23% had bipolar disorder rather than MDD [16]. Thus, there is a need to move beyond the assumption that women screening positive on the EPDS are a homogeneous “depressed” group. The same holds true for screening tools used for other diagnoses such as anxiety and psychosis. These limitations can be addressed by combining screening tools with clinician-based evaluations and diagnoses.

Other aspects that remain unclear in the clinical perinatal literature are the trajectories of different illnesses, the predictors of severe illness course, and whether treatment success is influenced by factors such as sociodemographic variables, comorbidities, medical and obstetrical complications and childhood adversity. Following patients longitudinally may facilitate answers to some of these questions.

In summary, there are still many unanswered questions about perinatal mental health in at least four broad areas: (1) the factors that differentiate between those with perinatal distress and those with more severe perinatal psychiatric disorders, (2) the course of different perinatal mental disorders, (3) the best treatments and practices across the spectrums of clinical severity and perinatal stage (pregnancy trimesters and postpartum period) for different disorders, and (4) the effects that sociodemographic variables have on treatment adherence and effectiveness.

These research questions have yet to be answered due to constraints of time, cost, along with patient and clinician burden. Researchers often encounter problems with recruitment and retention of participants, inadequate statistical power due to low incidence rates, and large variance in terms of treatments and treatment outcomes [17]. Because of these difficulties, and the challenges associated with studying pregnant women, many important research questions have been left unanswered, even though physicians are collecting rich data during their clinical appointments with patients.

Data collection that is integrated into a clinical setting, and facilitated by clinicians, offers an efficient and feasible way for overcoming these limitations. Using databanks for psychiatric research can be relatively inexpensive [18]; it has the potential to decrease cost and improve outcomes for patients and providers [19]. Creating such a mechanism at multiple clinical sites would allow for the accumulation of large sets of diverse data into a databank which can be used to study a large variety of research questions.

Such multisite clinical databanks have been created increasingly in the field of general perinatal health [20,21,22] and general psychiatry [23,24]. To our knowledge, however, there is a paucity of such databanks focused on women’s mental health and perinatal psychiatry. The utilization of multisite clinical databanks is an advantageous methodology because it allows health records to be analyzed on a numerically greater scale with more diverse samples, resulting in increased statistical power, generalizability, external validity and greater subtyping within heterogeneous diagnostic groups [25]. Additionally, they allow for increased collaboration between research sites while conducting longitudinal research, which results in more comparable results due to standardization of methods and minimization of potential differences between sites.

The aim of this project was to create a multicenter perinatal mental health databank, which is now successfully under way in Quebec. Data collection occurs during clinical appointments and includes both standardized measures and clinical diagnostic evaluations. This information is used immediately by doctors during the clinical appointment and later by researchers for study purposes. Data collection occurs at least twice in the perinatal period, which allows for the collection of longitudinal data and the pursuit of further clinical questions without overburdening patients. For those women who are initially seen during pregnancy, data are collected again at 6 weeks and between 4–6 months postpartum. Women who are initially assessed in the early postpartum period are reassessed again at 4–6 months postpartum. This paper describes the process of creating this databank so that it may be replicated at other sites, therefore facilitating a better understanding of perinatal mental health and leading to improved patient care.

## 2. Materials and Methods

### 2.1. Overview

In 2014, the Quebec Reproductive Psychiatry Network (QRPN) was established. It consists of clinicians and researchers across six sites in Montreal and Quebec City, which offer perinatal psychiatry services. Collectively, these perinatal clinics serve a population of over 25,000 deliveries per year and receive over 800 consults annually, and collect rich information during patient assessments, which are stored as documents in patients’ charts. However, the collected information is not standardized across sites. Additionally, these data, until now, were not accessible for research purposes due to the absence of a databank that respected patient confidentiality and was accessible to researchers. Thus, the network’s first major goal was to create a mechanism to collect standardized patient data for clinical and research purposes, such that information collected during clinical appointments could be stored both in patients’ medical charts and into a secure databank that protects patient confidentiality.

### 2.2. Standardizing the Collection of Clinical Information

From 2014–2016, regular meetings were held to determine the network’s broad areas of research interest and the relevant corresponding variables. A two-pronged approach was used to select the best measures for each outcome of interest. Searches were conducted through Medline and HAPI (Health and Psychological Instruments), as well as through the McGill University library website and public search engines (e.g., Google Scholar). Outcome measures and tools that appeared relevant were further examined for adequate psychometric properties. In a parallel iterative process, the network members discussed search results and provided input on their familiarity and experience with different measures, and their opinions regarding their practicality and ease of use. Additional questionnaires were created to address variables such as demographic characteristics and medical histories in order to standardize history-taking across sites. Every attempt was made to find the right balance between length/burden to the patient and inclusion of the most relevant information. Permission and scoring manuals were requested from authors when needed, and tools not available in French were translated and back-translated, as both French-speaking and English-speaking patients are served in Quebec.

### 2.3. Choosing the Software

Finally, because the network wanted to collect and store data electronically, electronic survey software programs were reviewed, and meetings were held with hospital information technology departments, as well as with software developers, to select the program best suited to the network’s needs. While choosing the software, the project needs were considered. The ideal software would have the following characteristics: the ability to collect data offline, function across the different sites, export data easily for analysis, and be linked to a data server located in Canada.

### 2.4. Ethical Standards

The databank mechanism was designed to be in accordance with prevailing ethical standards established in the 1964 Declaration of Helsinki and its later amendments and was reviewed by the Institutional Review Board of the primary investigating site using their Multicenter Mechanism (reference code: 17-115/MP-05-2018-774), which facilitates research by simplifying approval for collaborating sites. Written informed consent to have their data included in our databank is obtained from patients prior to their inclusion in the databank. Patients were assured that all data are automatically coded so that no identifying information enters the databank besides patients’ email addresses. The confidentiality section of the consent form states the following: “A separate list will be stored in the office of the Principal Investigator of this project, which links participants’ code numbers to their names and other identifying information. Email addresses in the databank will be heavily protected behind many layers of security provided by the software system and all email addresses will be removed from any analysis or exportation of data. Only specified members of the research team will be able to view your email address in the databank and will only do so when they need to contact you for future phases of the study. All the information collected during the research project will remain confidential to the extent provided by law. You will only be identified by a code number”.

## 3. Results

The process of initiating this collaboration, meeting, brainstorming, selecting the software, determining research areas of interest, and selecting outcomes and measures across different sites (described above) constitutes our methodological approach. Presently, we describe the final design of the databank (the “result” of this development process) in terms of the final research questions agreed upon, the measures included, and the process of data collection, as well as some preliminary demographic data. The scoring manual is provided in the Supplementary Materials in order to facilitate the adaptation and use of our protocol by others.

### 3.1. Questions

**Figure 1:**
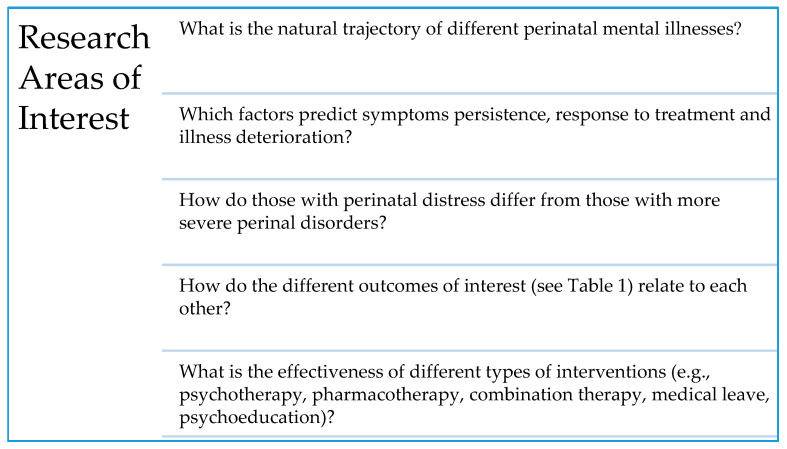
Research areas of interest.

The questions listed below represent the distillation of the broader research areas of interest; they were determined to be essential to the project and reflective of its primary purpose (Figure 1). These questions were selected because they were clinically relevant, have the potential to change current practice, and could be feasibly answered. It is expected that with time, more questions and measures will be added based on emerging trends that may suggest new hypotheses or questions. The databank was designed in such a way that researchers can identify a group of patients relevant to their query through the use of filters.

For example, a researcher interested in studying women whose babies were admitted to the neonatal intensive care unit (NICU) could easily find relevant data on this cohort by filtering for participants who had identified as such on their intake survey. If investigators want more information for their research, they can obtain approval from the Ethics Board to conduct a chart review of the hospital records. The same is possible in terms of posing questions regarding a subpopulation that received a certain intervention or diagnosis or medication class etc.

### 3.2. Justification of Variable Choice and Questionnaire Contents

Each research area of interest was broken down into relevant variables and outcome measures. The main variables of interest were as follows: maternal depression, anxiety, psychosocial risk, attachment, bonding and maternal functioning. These variables have been identified as risk factors in the literature, and are often associated with one another. In addition, they are relevant to the research areas of interest described above, which stem from both the literature and clinical practice.

Depression is estimated to affect roughly 13% of women globally, with a range of 5–25% [26]. Anxiety disorders have an even higher incidence than mood disorders [27], and it is believed that perinatal anxiety is underrecognized and undertreated compared to perinatal depression [28]. Moreover, the two disorders may be linked, as higher depression scores in early pregnancy may be associated with higher rates of anxiety later in pregnancy [29].

Psychosocial risk factors such as experiencing stressful life events during the perinatal period, lack of social support, and low socioeconomic status [30] have been linked to perinatal depression. Both mental health issues such as postpartum depression and psychosocial adversity such as stressful life events can lead to negative maternal and infant health outcomes [31,32,33,34].

Attachment style and bonding were included in our databank since the literature has long suggested that the quality of attachment may predispose to certain affective disorders [35]. Insecure attachment may increase the risk of developing mental disorders in general and mood disorders in particular [36]. Moreover, maternal attachment is important for the normal development of an infant’s social, cognitive and behavioral skills. Maternal depression has been associated with insecure and disorganized attachment [37] and more harsh or disrupted parenting behaviors, which may contribute to poor health outcomes later in childhood [38].

Attachment is an aspect of the relationship between a child and caregiver that is involved with making the child feel safe, secure and protected [39]. It refers to the child’s experience and feelings. Bonding, on the other hand, refers to the other direction of the mother–infant relationship, or the mother’s feelings and her subjective experience of her newborn. Mental disorders such as postpartum depression and posttraumatic stress disorder have been associated with impaired bonding [40], thus making it another important area to include in our research.

A summary of the relevant variables and outcome measures is shown in Table 1. Details of each measure’s psychometrics can be found in the Supplementary Material. Maternal functioning correlates with infant development [41,42,43], and has been found to be inversely related to maternal depression [41].

### 3.3. Agreed upon Study Setting and Sample

Participants in the databank are consenting women who were referred to one of the participating perinatal mental health services during pregnancy and up to 6 months postpartum. The ability to respond to questionnaires in either English or French is an inclusion criterion.

All patients arriving at their clinical assessments are asked to fill out questionnaires on a tablet device. The questionnaires and forms of consenting participants are exported to the secure server of the databank.

### 3.4. Final Procedures

Upon arrival at the clinic or prior to their appointments, patients are informed of the databank project by a clinician or research assistant. They are given an explanation of the rationale and procedures of the databank project and are asked to sign a consent form if they wish to participate. Due to the longitudinal nature of the study, and to facilitate the process of contacting patients, participants are also asked to provide contact information on a separate form, including their email address, phone number and the contact information of a relative.

This information is needed so that patients can be easily contacted throughout the study and sent surveys at the appropriate times. The participant’s email address is stored in the databank and categorized as an “identifier”. This allows it to be de-identified, separated from data, and made non-visible. All other contact information is not stored in the databank and is only accessible to those with specialized user rights (the project coordinator, the research coordinator, and the research assistants).

After providing this information, participants are asked to fill out a set of standardized measures and sociodemographic questions on a tablet device. Once patients finish completing the questionnaires, they meet with their clinician who has the opportunity to review and address relevant questionnaire results with them. Additionally, physicians fill out a standardized form on the tablet device which documents the participant’s current and past obstetrical, medical and psychiatric histories and their diagnoses according to the Diagnostic and Statistical Manual for Mental Disorders, fifth edition (DSM-V) as well as the treatment plan for the patient.

After the clinical appointment, all questionnaires and physician forms from the tablet are exported to the research databank. It is important to mention that as a result of the COVID-19 global pandemic, in-person recruitment has become limited at several sites, thus, online recruitment has been offered where needed.

### 3.5. Timeline

The number of times patients are assessed and the measures they fill out depends on the perinatal stage that they are in when first seen at the clinic. Participants who are first assessed during pregnancy fill out questionnaires during three separate assessment time points: at their first intake appointment, at 6 weeks postpartum and at 4–6 months postpartum. Participants who are first assessed postpartum fill out questionnaires only twice: at their initial appointment and at 4–6 months postpartum. Although the first data collection time point always occurs in tandem with an actual clinical assessment, the second and third data collection may occur by phone, or online, depending on the patient’s preference. Due to the impact of COVID-19 pandemic, some centers opted for online recruitment and data collection for all visits in accordance with health regulations. Online surveys are sent securely by email from the databank online software. Details on the timeline of protocols are shown in Table 2 and Table 3.

### 3.6. Software

After a review of software options, a secure web application called REDCap (Vanderbilt University, Nashville, USA) was chosen because it best fit the needs we described above. REDCap is used to make surveys and allows for data entry and seamless export of data into different statistical programs. REDCap is recognized for outstanding protection of confidentiality of identifiable data, easy handling of longitudinal data, and the ability to link-up with other data sources (i.e., medical charts). To protect patient confidentiality, REDCap provides detailed audit trailing, record-locking, fine-grained control over user rights and de-identification functions for data export. REDCap is compliant with Health Insurance Portability and Accountability Act (HIPAA), and it is highly secure [59]. Certain fields in REDCap can be marked as “identifiable”, thus allowing researchers to de-identify data during export. All data are automatically coded so that no identifying information enters the databank besides patients’ email addresses. Before clinical appointments, REDCap is used to administer surveys to patients on tablets. After the appointments, data from the tablet are securely transmitted to and from the REDCap server that is located in Canada, meets Canadian Privacy Standards and is managed by the Information Management Service at the central clinical site.

### 3.7. Use of the Data

To date, 225 records have been collected in the databank, of which 175 are completed. Of the 225 participants, 167 began participating during pregnancy, while 57 began participating postpartum. The response rate for participants at 6-week follow up from all sites is 71.4%. Response rate for participants at 4–6 month follow up from all sites is 77.2%. The mean age of participants is 32 years, and some demographics are shown in the table below (Table 4).

The majority of participants reported that the questionnaire was easy or very easy to complete (78.9%), and 84.3% reported no or little discomfort in relation to answering the questions. For example, four participants commented on the Postpartum Bonding Questionnaire items 18 and 24 that explore feelings of harming the baby; it made them uncomfortable or sad to think that some mothers faced having such thoughts. Clinicians using the questionnaire generally report they found it user friendly.

## 4. Discussion

### 4.1. Summary

This paper describes the formation and functioning of a multisite databank that collects medical and psychosocial variables from pregnant and postpartum patients. Data collection is focused on maternal depression, anxiety, psychosocial risk, attachment, bonding and maternal functioning, and is underway with 225 cases enrolled to date.

### 4.2. Databank Research Areas in Psychiatry

Clinical research that utilizes computerized databases has greatly enhanced many areas of knowledge in psychiatry [60]. An international review found that database research in psychiatry has included areas such as pharmacoepidemiology, perinatal, etiological, suicidology and health service research [18]. Databanks focused on pregnancy and mental illness are fewer than those related to pharmacology, suicidology and other areas, highlighting a need for more databanks focused on reproductive and perinatal psychiatry.

### 4.3. Perinatal Databanks

Psychiatry databanks related to illness and pregnancy include the General Practice Research Database (GPRD): UK, Denmark’s Psychiatric Register, and Denmark’s Birth & Congenital Malformation Registers. These databases have been used to explore fertility, stillbirth rate, perinatal death rate, and other pregnancy outcomes in women with psychotic disorders [18].

Another rich source of ongoing research is the Pregnancy Risk Assessment Monitoring System (PRAMS) in the United States, which is a surveillance system of maternal behaviors, attitudes, and experiences in the perinatal period [58]. It provides ongoing state-based data for key maternal and child health indicators over time. Most of the core topics covered relate to general and obstetric health rather than mental health (e.g., multivitamin use, oral health, and contraception use), but postpartum depressive symptoms are also one of the core topics of interest in this surveillance system. Our databank collects some obstetric and medical data, but focuses more on mental health outcomes, such as depression, anxiety, psychosocial risk and relationships.

The databanks described above are mostly national registries that have governmental support. Our databank is center-based and much smaller in scope. Hospital or center-based research tends to be smaller than governmental registries, yet can conduct more detailed and highly specialized research programs. For example, the Center for Women’s Mental Health at the Massachusetts General Hospital has both a Clinical and a Research Program that address women’s mental health issues across the lifespan. Their research is regularly published in scientific journals. Current active research programs that focus on the perinatal period include the Massachusetts General Hospital Postpartum Psychosis Project, the national pregnancy registry for psychiatric medications, the UPWARD study (Understanding and Preventing Women’s Relapse of Depression) and the UPWARD(S) study which looks at suicidal thoughts and behaviors in peripartum women [61].

### 4.4. Limitations

Our databank has several limitations. The numbers recruited to date remain small, and the buildup of cases will take time. It is known that amongst the different types of databanks, those based on clinical interviews and surveys are not considered the best way to acquire large numbers [62]. This is somewhat offset by the depth and quality of information obtained by physician evaluation and interviews combined with validated screening tools.

Databases are often used in psychiatry research by linking clinical and national administrative databases [18]. Linking the data we collected to patients’ clinical records would have made it possible to broaden the scope of research. An example of record linkage research is Sweden’s population-based registers that were used to link reports of suicide to psychiatric diagnoses and unemployment [62]. However, this was not possible in our databank due to confidentiality, ethical and technical reasons. Linking our databank with medical records in the future would enable us to answer many more research questions related to medical illness, infant and child outcomes, and many other outcomes.

### 4.5. Strengths

Despite these limitations, this type of databank offers an advantage to most databank-related research. Its data stem from well-chosen outcome measures selected to answer specific research questions. This contrasts with most database research, which draws on the type of data that are gathered for administrative reasons and are readily available for research, which limits the research questions asked. These questions may be opportunistic, based on available data, and may not reflect the priorities of clinicians or patients [18].

For example, medication-oriented research may be one of the most common types of research due to the presence of accessible readily available data on medication prescription [62]. In addition, proxy measures are often used in this type of research [18,62]; for example, prescriptions may be used to indicate medication use despite the fact that medications may not have been dispensed or used with adherence.

Reviews on databank research point to the need for a transition whereby databanks are designed to reflect research priorities, which should be in turn a reflection of clinical needs. [18,62]. Our databank addresses this need, as the research areas/questions were formulated first and the outcome measures chosen carefully. Thus, the data collected are tailored to accurately answer research questions that are important and not easily answered by the usual databank research available.

### 4.6. Challenges

Outlined below are some of the specific problems encountered by our network and some useful approaches and solutions to them. In the absence of guiding literature on how to create a multicenter electronic databank in perinatal psychiatry, this methodological paper may prove useful to others in the field.

This databank involves several researchers with overlapping but differing research interests. Initial brainstorming meetings resulted in many interesting research questions and variables. It was important to find the balance between collecting rich data and keeping the protocol realistic, feasible and not too burdensome for both clinicians and patients. It was thus important to reach a consensus about the main aims of the databank and then to select the most essential variables to formulate key research questions.

Methods were developed to maximize data collection without increasing the length of the questionnaires. The demographic questionnaire that the patient completes contains branching logic, for example, to only ask about number of children at home if a patient has indicated that she has children. To minimize the length of the standardized form that the clinicians fill out, branching logic was added to several sections, for example, to display only relevant information for specific DSM-V diagnoses, and to make visible fertility treatment options only if the patient indicates an infertility diagnosis. For secondary questions that interested one researcher or site (e.g., types of fetal abnormalities), a filtering system was instituted, whereby a researcher is able to search and filter for patients that answered positively to “abnormality on neonatal testing” in the databank, and then conduct a chart review after obtaining ethics approval for this subproject.

Agreeing on the appropriate instrument for every outcome and reviewing the literature on each was time consuming but necessary. It was useful to write and share a written review of the pros, cons, psychometrics and validation studies of the instruments while selecting the measurements of choice.

Perhaps one of the longest but most fruitful processes was the development of a standardized form that physicians fill out during clinical appointments. This ensures that the physician history-taking, diagnosis and treatment plan are standardized and thorough, and will translate into retrievable data across sites. The feasibility and acceptability of this tool may be evaluated in the future and may be modified to other research programs and specialties.

Another challenge that may be faced when setting up electronic databanks is identifying the right software. It is advisable for researchers to outline their unique requirements (e.g., need for exporting data into certain programs, tablets for filling-out surveys, offline capabilities for data collection etc.) and to meet early on with their institution’s information technology, medical records and ethics departments to understand their requirements and research software options.

Finally, when conducting research across many sites, obtaining ethics approval can be difficult. Some institutions may have a multicenter mechanism, which facilitates research across sites by allowing one site’s ethics review board to act as the primary ethics review board and be recognized by other sites, thus minimizing repetitive paperwork and addressing only site-specific concerns.

## 5. Conclusions

This paper describes the process of creating an electronic multicenter databank for perinatal psychiatry in the province of Quebec. Such a databank may be replicated by other centers to facilitate research that can advance the understanding of mental health issues of mothers, and thus inform best practice and policy formation with regard to this population.

## Figures and Tables

**Table 1:** Outcomes and measures.

Variable	Measure/Tool	Reliability	Validity
Demographic characteristics	Demographic form (Tool created by QRPN)	N/A	N/A
Medical, obstetric and psychiatric history	Physician Form (Tool created by QRPN)	N/A	N/A
Depression	Edinburgh Postnatal Depression Scale [44,45]	Internal consistency: α = 0.88 [44]	Sensitivity: 63–93% Specificity: 88–99% [15]
Anxiety	Generalized Anxiety Disorder-7 [46]	Internal consistency: α = 0.92 [46]	Sensitivity: 89% Specificity: 82% [46]
	Cambridge Worry Scale [47]	Internal consistency: α = 0.79	Correlation between trait anxiety and total worry scores (r = 0.45–0.56)
Psychosocial risk	Antenatal Risk Questionnaire [48]	N/A	Sensitivity: 62% Specificity: 64%
	Postnatal Risk Questionnaire [49]	N/A	N/A
Adult peer relationship style	Relationship Questionnaire [50]	Test–retest: r = 0.39 to 0.58 [51]	Convergent validity with interview attachment ratings: r = 0.22 to 0.50 [52]
Childhood adversity	Adverse Childhood Experiences Questionnaire [53]	Internal consistency: α = 0.88 [54]	Convergent validity with adult attachment interview: chi sq = 17.50, df = 4, *p* = 0.002 [54]
Bonding	The Postpartum Bonding Questionnaire [55]	Test–retest: Scale 1, r = 0.95 Scale 2, r = 0.95 Scale 3, r = 0.93 Scale 4, r = 0.77 [55]	* Sensitivity: Scale 1 = 82% Scale 2 = 67 to 88% [56]
Maternal Functioning	Barkin Index of Maternal Functioning [3,41,57]	Internal consistency: α = 0.87 [57]	Construct validity: Significant correlations found with three relevant measures [57]
	Pregnancy Risk Assessment Monitoring System Questionnaire (PRAMS) [58]	N/A	N/A

QRPN: Quebec Reproductive Psychiatry Network; * Scale 3 and 4 had unsatisfactory validity but were deemed useful for clinical purposes.

**Table 2:** Timeline of protocol 1 (first seen during pregnancy).

Measure	Intake	~6 Weeks Post-Partum	4–6 Months Post-Partum
Contact information and demographics—full version	✓		
Exercise	✓	✓	✓
Complementary alternative medicine (CAM)	✓		
Newborn and birth information		✓	
Demographics—update		✓	✓
The Edinburgh Postnatal Depression Scale (EPDS)	✓	✓	✓
The Generalized Anxiety Disorder (GAD)-7	✓	✓	✓
The Antenatal Risk Questionnaire (ANRQ)—full version	✓		
Postnatal Risk Questionnaire (PNRQ)		Last 3 questions ✓	Full version ✓
The Cambridge Worry Scale (CWS)	✓		
The Relationship Questionnaire (RQ)	✓		
The Adverse Childhood Experiences Questionnaire (ACE)	✓		
The Postpartum Bonding Questionnaire (PBQ)		✓	✓
Pregnancy Risk Assessment Monitoring System Questionnaire (PRAMS)—14 questions		✓	✓
The Barkin Index of Maternal Functioning (BIMF)		✓	✓
Physician history-taking form	✓		
Compliance with recommendations		✓	✓
COVID-19 module	✓		

**Table 3:** Timeline of protocol 2 (first seen after pregnancy).

Measure	Intake	4–6 Months Post-Partum
Contact information and demographics—full version	✓	
Exercise	✓	✓
Complementary alternative medicine (CAM)	✓	
Newborn and birth information	✓	
Demographics—update		✓
The Edinburgh Postnatal Depression Scale (EPDS)	✓	✓
The Generalized Anxiety Disorder (GAD)-7	✓	✓
Postnatal Risk Questionnaire (PNRQ)—full	✓	✓
The Relationship Questionnaire (RQ)	✓	
The Adverse Childhood Experiences Questionnaire (ACE)	✓	
The Postpartum Bonding Questionnaire (PBQ)	✓	✓
Pregnancy Risk Assessment Monitoring System Questionnaire (PRAMS)—14 questions only	✓	✓
The Barkin Index of Maternal Functioning (BIMF)	✓	✓
Physician history-taking form	✓	
Compliance with recommendations		✓
COVID-19 module	✓	

**Table 4:** Demographics of current participants.

Demographic Features	Number (Percentage)	
Single (never married)	18 (10.2)	176 (Missing = 0)
Married	76 (43.2)	
Living with your partner	78 (44.3)	
Divorced/separated	4 (2.3)	
Widow	0 (0.0)	
Unemployed	110 (62.9)	175 (Missing = 1)
Employed	65 (37.1)	

## Supplementary Materials

The following is available online at https://www.mdpi.com/1660-4601/17/24/9352/s1, Scoring Manual.
