# Supplementary material for: Creating a Multisite Perinatal Psychiatry Databank: Purpose and Development
Source: Int J Environ Res Public Health. 2020 Dec 14;17(24):9352. doi: 10.3390/ijerph17249352 (PMC7765035; doi:10.3390/ijerph17249352)
Supplement: Supplementary file 1 [file ijerph-17-09352-s001.pdf]

Scoring Manual for Perinatal Multisite Databank Project

Contents

Demographics 2
Edinburgh Postnatal Depression Scale (EPDS) 2
Generalized Anxiety Disorder 7-Item (GAD-7) Scale 3
Antenatal Risk Questionnaire (ANRQ) 4
Postnatal Risk Questionnaire (PNRQ) 5
The Cambridge Worry Scale (CWS) 6
Relationship Questionnaire (RQ) 7
Adverse Childhood Experience (ACE) Questionnaire 8
Postpartum Bonding Questionnaire/Instrument (PBQ) 9
Pregnancy Risk Assessment Monitoring System Questionnaire (PRAMS) 12
Barkin Index of Maternal Functioning (BIMF) 12
Physician History-Taking Form (PHTF) 14
Quick Sheets for Physicians to Interpret Scores of Measures on Redcap 15

Demographics

Developed by the team.

DESCRIPTION OF MEASURE: basic demographic information including birthday, marital status, household information, employment, immigration status, religion, ethnicity, language, income and child protective services history.

SCORING: None.

Edinburgh Postnatal Depression Scale (EPDS)

CONSTRUCT MEASURED: Maternal Depression.

SCORES OBTAINED: Not at risk or at risk for depression (dichotomous outcome).

DESCRIPTION OF MEASURE:

- This 10-item scale designed to screen for postpartum depression in community samples has also been used in follow up studies up to 4 years postpartum.
- Questions refer to symptoms experienced in the past 7 days.

SCORING:

- The items are rated on a scale from 0 to 3 (response schemes vary by question).
- Items 3, 5–10 are reverse scored (3 to 0).
- Sum scores for the 10 items to generate a total score.

THEORETICAL RANGE: Raw Scores from 0 to 30.

INTERPRETATION OF SCORING / CUT-OFF:

- Higher score indicates more severe depressive symptoms.
- Total score of 13 or higher are in clinical range, at risk for depressive disorder.
- Item 10 *(The thought of harming myself has occurred to me)*: any score greater than 0 should be evaluated for safety of the mother and baby.

RELIABILITY:

- The split-half reliability of the scale is 0.88, and the standardized alpha coefficient is 0.87.

VALIDITY:

- In validation studies, using a cut score of 12, the EPDS was found to have a sensitivity of 68% to 95%, and a specificity of 78% to 96% when compared to a diagnosis of major depression made by psychiatric interview.

REFERENCES:

Cox, J.L., Chapman, G., Murray, D., & Jones, P. (1996). Validation of the Edinburgh Postnatal Depression Scale (EPDS) in non-postnatal women. *Journal of Affective Disorders, 39*, 185-189.

Cox, J.L., J.M. Holden, and R. Sagovsky, *Detection of postnatal depression: development of the 10-item Edinburgh Postnatal Depression Scale.* The British Journal of Psychiatry, 1987. **150**: p. 782-786.

Generalized Anxiety Disorder 7-Item (GAD-7) Scale

CONSTRUCT MEASURED: Maternal anxiety.

SCORES OBTAINED: Level of Maternal anxiety.

DESCRIPTION OF MEASURE:

- 7 item measure; time frame refers to the last two weeks.
- First 2 items on the GAD assess core anxiety symptoms (scores on the GAD-2 subscale range from 0 to 6).
- The items are rated on a scale from 0 to 3 (0 being “Not at all” to 3 being “Nearly every day” to describe how often one experiences symptoms of anxiety).

SCORING:

Scores are summed for all responses.
0 = not at all.
1 = several days.
2 = more than half the days.
3 = nearly every day.

THEORETICAL RANGE:

- Theoretical range: 0–21.

INTERPRETATION OF SCORING / CUT-OFF

- Higher scores = more symptoms of anxiety.
- Total scores of 5, 10, and 15 represent mild, moderate, and severe anxiety symptoms, respectively.

RELIABILITY:

- Excellent internal consistency (Cronbach alpha coefficient = 0.92)
- Test–retest reliability also good (r=0.83).

VALIDITY:

- Good convergent validity with the Beck Anxiety Inventory (r=0.72) and the anxiety subscale of the Symptom Checklist-90 (r= 0.74).

REFERENCES

Kroenke, K., Spitzer, R.L., Williams, J.B.W. Monahan, P.O., & Lowe, B. (2007). Anxiety disorders in primary care: prevalence, impairment, comorbidity, and detection. *Annals of Internal Medicine, 146*, 317-325.

Antenatal Risk Questionnaire (ANRQ)

CONSTRUCT MEASURED: Maternal prenatal psychosocial risk (for developing postnatal depression).

SCORES OBTAINED: Levels of maternal prenatal psychosocial risk.

DESCRIPTION OF MEASURE:

- 12 scored items with additional unscored items.
- Measures maternal prenatal psychosocial risk.
- Used to assess the presence and the perceived impact of nine psychosocial risk variables: a) perceived emotional support from mother as a child; b) history of low mood, mental illness and treatment received; c) perceived level of emotional support from partner; d) life stresses in the last 12 months; e) tendency to worry; f) need for order; g) level of social support; h) history of emotional abuse; g) history of physical or emotional abuse.
- Shortened measure derived from the 17-item Pregnancy Risk Questionnaire (PRQ).
- ANRQ is a selection of PRQ items that showed the highest associations with later onset of postnatal depression.

SCORING:

- Higher score = greater risk.
- Questions 1, 1b, 3, 7 and 8 are scored 5 for “yes” and 0 for “no”.
- Score questions 1a, 2, 3a, 4, 5, 6, and 9 as the number circled.
- Sum all scored questions (yes/no and circled answers).
    - Note: Questions and sub-questions under 1b and question 1c are unscored.

THEORETICAL RANGE: 7–60.

INTERPRETATION / CUT-OFF:

- Increased psychosocial risk if <u>any</u> of the following criteria are present:
    - Total score at or above **23.**
    - Q1 (Have you ever had a period of 2 weeks or more when you felt particularly worried, miserable or depressed?) = yes <u>**AND**</u> either (1) Q1a is greater than or equal to 4 (interfering with work or relationships with family and friends) OR (2) Q1b = yes (seeking professional help).
- History of abuse: if Q7 = yes or Q5 = yes.

SENSITIVITY:

- Using the cut-off of 23, this scale has good sensitivity (0.62) and specificity (0.64). Predictive value of 0.3 for postnatal depression.

VALIDITY: Has been validated with pregnant women.

REFERENCES:

Priest, S.R., Austin, M.P., Barnett, B.B., & Buist, A. (2008). A psychosocial risk assessment model (PRAM) for use with pregnant and postpartum women in primary care settings. *Arch Womens Ment Health, 11*, 307-317.

Austin, M. P., Colton, J., Priest, S., Reilly, N., & Hadzi-Pavlovic, D. (2013). The antenatal risk questionnaire (ANRQ): acceptability and use for psychosocial risk assessment in the maternity setting. Women Birth, 26(1), 17-25. doi:10.1016/j.wombi.2011.06.002

Austin, M.P. (2004, updated 2017). Antenatal Risk Questionnaire. Provided by email (2018): M.AUSTIN@UNSW.EDU.AU

Postnatal Risk Questionnaire (PNRQ)

CONSTRUCT MEASURED: Maternal postpartum psychosocial risk.

SCORES OBTAINED: Identify risk factors associated with the development of perinatal depression.

DESCRIPTION OF MEASURE:

- 12 scored items with extra unscored questions.
- Used to identify risk factors associated with the development of postpartum depression.

SCORING:

- The PNRQ is identical to the ANRQ with an additional 3 post-natal questions. Since the PNRQ has never been fully validated, the 3 postnatal items (last 3 questions) are unscored and just used as additional information for clinicians. Therefore, the PNRQ uses the same scoring as the ANRQ.
- Higher score = greater risk.
- Questions 1, 1b, 3, 7 and 8 are scored 5 for "yes" and 0 for "no".
- Score questions 1a, 2, 3a, 4, 5, 6, and 9 as the number circled.
- Sum all scored questions (yes/no and circled answers).
    - Note: Questions and sub-questions under 1b and question 1c are unscored.

THEORETICAL RANGE: 10–77.

INTERPRETATION OF SCORING / CUT-OFF:

- Increased psychosocial risk if <u>any</u> of the following criteria are present:
    - Total score at or above**24.**
    - Q1 (Have you ever had a period of 2 weeks or more when you felt particularly worried, miserable, or depressed?) = yes **<u>AND</u>** either (1) Q1a is greater than or equal to 4 (interfering with work or relationships with family and friends) OR (2) Q1b = yes (seeking professional help).
    - History of abuse: if Q7 = yes or Q5 = yes.

RELIABILITY: Logistic regression of PNRQ responses and MINI diagnostic interview showed significant associations between high trait anxiety, emotional childhood abuse, frightening/disappointing birth experience and psychosocial risk measured by the PNRQ. For women who responded "yes" to question 1b (previous mental health problems that required professional help) and/or reported interference with work/relationship (>3 on the Likert scale in question 1a), they were more likely to meet diagnostic criteria for a previous mental health episode (MINI).

VALIDITY: Tested in mothers with infants up to 12 months of age, but this scale has never been fully validated.

REFERENCES:

Christl, B., Reilly, N., Smith, M., Sims, D., Chavasse, F., & Austin, M.-P. (2013). The mental health of mothers of unsettled infants: is there value in routine psychosocial assessment in this context? Arch Womens Ment Health, 16(5), 391-399. doi:10.1007/s00737-013-0360-0

The Cambridge Worry Scale (CWS)

CONSTRUCT MEASURED: Maternal Worry

SCORES OBTAINED: Content and extent of maternal worries

DESCRIPTION OF MEASURE:

- 16 item scale, time frame refers to how participant feels "currently".
- Examines women's concerns about the health of their baby within the context of other concurrent worries, both pregnancy related and more general.
- Open-ended question at the end allows respondents to list experienced worries not on the list.
- Responses are made on a 6-point Likert-type scale (0 "not a worry" to 5 "extremely worried").

SCORING:

- Scores are obtained from summing all the answers.
    - 0 = not a worry to 5 = extremely worried about.
- Sub scales include socio-economic (#1,2,8) health (6,7,9,16) socio-medical (# 10–13) and relationships (# 4,5).

THEORETICAL RANGE: 0–80**.**

INTERPRETATION / CUT-OFF:

- Higher score = more distress.
- No cut-off score at this time.

RELIABILITY:

- Scale exhibits satisfactory internal consistency (Cronbach alpha coefficient = 0.79).
- Good test–retest correlations (r = 0.69 to 0.72).
- Substantial correlation between trait anxiety and total worry scores (r = 0.54).

VALIDITY:

- There was substantial co-variation between trait anxiety and the total worry scores at each time (time 1 r (1165) = 0.54; time 2 r (1165) = 0.44; time 3 r (1137) = 0.46; all significant p <.000).

REFERENCES:

Green, J.M., Kafetsios, K., Statham, H.E., & Snowdon, C.M. (2003). Factor structure, validity, and reliability of the Cambridge Worry Scale in a pregnant population. *Journal of Health Psychology, 8*, 753-764.

Relationship Questionnaire (RQ)

CONSTRUCT MEASURED: Adult–peer relationship style.

SCORES OBTAINED: Adult–peer relationship style assessed.

DESCRIPTION OF MEASURE:

- Adult–peer relationship style is assessed.
- Four relationship categories: (A) secure, (B) fearful, (C) preoccupied, (D) dismissing
- 2 item measure:
    - First item made up of four short paragraphs, each describing a prototypical attachment pattern as it applies to close adult peer relationships. Respondents are asked to select the pattern that best describes their close peer relationships.
    - Second item asks participants to rate their degree of correspondence to each relationship prototype on a 7-point Likert-type scale (1 “not at all like me” to 7 “very much like me”).

SCORING:

- Category most like participant is represented by a letter (A,B,C,D) and then rated from 1 to 7 for each of these (1 = not at all like me, 4 = somewhat like me, 7 = very much like me):
    - ***Secure:*** It is easy for me to become emotionally close to others. I am comfortable depending on them and having them depend on me. I don't worry about being alone or having others not accept me.
    - ***Fearful:*** I am uncomfortable getting close to others. I want emotionally close relationships, but I find it difficult to trust others completely, or to depend on them. I worry that I will be hurt if I allow myself to become too close to others.
    - ***Preoccupied:*** I want to be completely emotionally intimate with others, but I often find that others are reluctant to get as close as I would like. I am uncomfortable being without close relationships, but I sometimes worry that others don't value me as much as I value them.
    - ***Dismissing:*** I am comfortable without close emotional relationships. It is very important to me to feel independent and self-sufficient, and I prefer not to depend on others or have others depend on me.

THEORETICAL RANGE: 4–28 (note: the total score has no meaning); 1–7 for each item.

INTERPRETATION / CUT-OFF:

- The highest of the four attachment prototype ratings can be used to classify participants into an attachment profile/category.
- Answer to forced-choice item (Q1) important when participants rate two or more attachment prototypes equally high (Q2).

RELIABILITY:

- Alpha coefficients were computed to assess the reliability of the prototype ratings. The reliabilities ranged from 0.87 to 0.95.

VALIDITY: Construct validity acceptable (See Guedeney et al. for additional details).

REFERENCES:

Bartholomew, K. & Horowitz, L.M. (1991). Attachment styles among young adults: a test of a four-category model. *Journal of Personality and Social Psychology, 61*(2), 226-244.

Guedeney, N., Fermanian, J., & Bifulco, A. (2010). [Construct validation study of the Relationship Scales Questionnaire (RSQ) on an adult sample]. *Encephale, 36*(1), 69-76. doi:10.1016/j.encep.2008.12.006

Adverse Childhood Experience (ACE) Questionnaire

CONSTRUCT MEASURED: Adverse childhood experiences.

SCORES OBTAINED: Level of childhood abuse and/or dysfunction in childhood household.

DESCRIPTION OF MEASURES:

- 10 items.
- Questions regarding childhood abuse (physical, psychological, sexual) and dysfunction in household (substance abuse, mental illness, mother treated violently, criminal behavior in household).
- Asks about experiences before the age of 18.

SCORING:

- For each item, yes is scored as 1 and no is scored as 0. Each item is added together to find the total score.
- Higher scores mean greater trauma exposure.

THEORETICAL RANGE: 0–10.

INTERPRETATION OF SCORING/CUT-OFF: None currently.

RELIABILITY: None currently.

VALIDITY: None currently.

REFERENCES:

Felitti, V. J., Anda, R. F., Nordenberg, D., Williamson, D. F., Spitz, A. M., Edwards, V., Koss, M. P., Marks, J. S. (January 01, 1998). Relationship of Childhood Abuse and Household Dysfunction to Many of the Leading Causes of Death in Adults:The Adverse Childhood Experiences (ACE) Study. *American Journal of Preventive Medicine, 14,* 4, 245-258.

Postpartum Bonding Questionnaire (PBQ)

CONSTRUCT MEASURED: Mother–infant bonding.

SCORES OBTAINED: Mother–infant bonding assessed over 4 domains.

DESCRIPTION OF MEASURE: Mother–infant bonding assessed over 4 domains: generally weak bonding**,** rejection/pathological anger**,** infant-focused anxiety**,** incipient abuse. Participants are asked to respond in a way "which seems right in your recent experiences".

SCORING: 6-point Likert scales (always, very often, quite often, sometimes, rarely, and never). These are divided into 4 factors which total scores for each factor. See following page for which items belong to each factor and whether they are reverse scored.

- Factor 1:
    - Purpose: general factor, identifies if there is a problem with the mother–infant relationship.
    - 12 items.
- Factor 2:
    - Purpose: identify severe infant–mother relationship disorders (e.g., bonding disorders, threatened rejection, established rejection, and pathological anger).
    - 7 items.
- Factor 3:
    - Purpose: infant-focused anxiety.
    - 4 items.
- Factor 4:
    - Purpose: identification of dangerous mothers.
    - 2 items.
    - Note: this factor has very low sensitivity (0.13 for pathological anger and 0.20 for severe anger).
- Total score: the authors of the scale note this can be used, however, looking at individual factor totals may be more useful. This has also not been validated in an independent sample.

	Factor	Scoring	Statement	Always	Very often	Quite often	Some-times	Rarely	Never
1	1	0 → 5	I feel close to my baby						
2	1	5 → 0	I wish the old days when I had no baby would come back						
3	2	5 → 0	I feel distant from my baby						
4	2	0 → 5	I love to cuddle my baby						
5	2	5 → 0	I regret having this baby						
6	1	5 → 0	The baby does not seem to be mine						
7	1	5 → 0	My baby winds me up						
8	1	0 → 5	I love my baby to bits						
9	1	0 → 5	I feel happy when my baby smiles or laughs						
10	1	5 → 0	My baby irritates me						
11	2	0 → 5	I enjoy playing with my baby						
12	1	5 → 0	My baby cries too much						
13	1	5 → 0	I feel trapped as a mother						
14	2	5 → 0	I feel angry with my baby						
15	1	5 → 0	I resent my baby						
16	1	0 → 5	My baby is the most beautiful baby in the world						
17	1	5 → 0	I wish my baby would somehow go away						
18	4	5 → 0	I have done harmful things to my baby						
19	3	5 → 0	My baby makes me feel anxious						
20	3	5 → 0	I am afraid of my baby						
21	2	5 → 0	My baby annoys me						
22	3	0 → 5	I feel confident when caring for my baby						
23	2	5 → 0	I feel the only solution is for someone else to look after my baby						
24	4	5 → 0	I feel like hurting my baby						
25	3	0 → 5	My baby is easily comforted						

THEORETICAL RANGE:

- Factor 1: 0–60.
- Factor 2: 0–35.
- Factor 3: 0–20.
- Factor 4: 0–10.
- Total score: 0–125.

INTERPRETATION OF SCORING/CUT-OFF:

- Factor 1 cut off: 11 = high (below 12 is normal).
- Factor 2 cut off: 16 = high (below 17 is normal).
- Factor 3 cut off: 9 = high (less than 10 is normal).
- Factor 4 cut off: 2 = high (less than 3 is normal).
- Total score:
  - Cut off: 26 = above 26 suggests some kind of disorder.
  - Cut off for 40 = cut-off for severe disorders.

VALIDITY:

- Factor 1:
  - Specificity (to identify normal mothers): 0.68–0.85.
  - To identify any disorder of the mother–infant relationship:
    - Sensitivity: 0.82–0.93.

        - Positive predictive value: 0.76.
    - To identify rejecting mothers:
        - Sensitivity: 0.92–1.0.
        - Positive predictive value: 0.23–0.46.
    - To identify dangerously angry mothers:
        - Sensitivity: 0.93.
        - Positive predictive value: 0.19.
- Factor 2:
    - Specificity (to identify normal mothers): 0.95–1.0.
    - To identify rejecting mothers:
        - Sensitivity: 0.68–0.88.
        - Positive predictive value: 0.45–0.76.
    - To identify dangerously angry mothers:
        - Sensitivity: 0.67.
        - Positive predictive value: 0.3.
- Factor 3:
    - Specificity (to identify normal mothers): 0.64.
    - To identify established rejection:
        - Sensitivity: 0.82.
    - To identify infant-focused anxiety in mothers:
        - Sensitivity: 0.61.
- Factor 4:
    - To identify moderate to severe anger in mothers:
        - Sensitivity: 0.16–0.20.
        - Positive predictive value: 0.5–0.78.
- Total score:
    - Cut-off of 26:
        - Specificity: 0.61.
        - Sensitivity: 0.84.
        - Positive predictive value: 0.79.
    - Cut-off of 40:
        - Specificity (of normal mothers): 0.89.
        - Sensitivity of rejecting mothers: 0.89.
        - Sensitivity of angry mothers: 0.80.

VALIDITY: Scale was validated in mothers who were evaluated using diagnostic interviews. For results see reliability

REFERENCES:

Brockington, I. F., Fraser, C., & Wilson, D. (2006). The Postpartum Bonding Questionnaire: a validation. *Arch Womens Ment Health, 9*(5), 233-242. doi:10.1007/s00737-006-0132-1

Brockington, I. F., Oates, J., George, S., Turner, D., Vostanis, P., Sullivan, M., . . . Murdoch, C. (2001). A Screening Questionnaire for mother-infant bonding disorders. Arch Womens Ment Health, 3(4), 133-140. doi:10.1007/s007370170010

Pregnancy Risk Assessment Monitoring System Questionnaire (PRAMS)

CONSTRUCT MEASURED: Presence of risk factors in the 12 months leading up to birth.

SCORES OBTAINED: N/A.

DESCRIPTION OF MEASURE: Stressful life events in the mother during the 12 months before her baby is born (e.g., loss of employment, financial stress, interpersonal conflict). This is part of a larger questionnaire and we are only using question 43 of the 2016 version of the PRAMS.

- 14 items or risk factors to which the participant responds yes/no.

SCORING: Each item lists a stressful life event that the respondent indicates if they experienced it or not (yes/no). There is no total score.

THEORETICAL RANGE: N/A.

INTERPRETATION OF SCORING/CUT-OFF: N/A.

RELIABILITY: N/A because this is just one question on a much larger measure.

VALIDITY: N/A because this is just one question on a much larger measure.

REFERENCES:

Centers for Disease Control and Prevention (2016) Pregnancy Risk Assessment Monitoring System www.cdc.gov/PRAMS/

Barkin Index of Maternal Functioning (BIMF)

CONSTRUCT MEASURED: Measuring of functioning of mothers during the first year of their infant's life.

SCORES OBTAINED: Level of maternal functioning.

DESCRIPTION OF MEASURE:

- 20 items.
- Asks how participant has been feeling over the past 2 weeks in the domains listed below.
- 6-point Likert scale (strongly disagree, disagree, somewhat disagree, neutral, somewhat agree, agree, and strongly agree).

SCORING:

- Total score by adding 20 items together:
    - Strongly disagree: 0.

- Disagree: 1.
- Somewhat disagree: 2.
- Neutral: 3.
- Somewhat agree: 4.
- Agree: 5.

- Items 16 and 18 are reverse scored.

THEORETICAL RANGE: 0 to 120.

INTERPRETATION OF SCORING / CUT-OFF

- Higher scores indicate greater level of functioning with a score of 120 representing optimal functioning.
- No clinical cut-off or threshold established.

Item-domain mapping: This is not used for scoring, just as a description of the components of the measure.

Functional area	*BIMF Item*
Self care	2, 11, 13
Infant care	12, 14
Mother–child interaction	4, 5, 15
Psychological well-being	1, 2, 3, 5, 7, 10, 11, 16, 18, 20
Social support	6, 8, 9
Management	7, 11, 13, 14, 17, 18
Adjustment	17, 19

RELIABILITY:

- Cronbach's alpha (measure of internal consistency): 0.87 (good) (replicated in a second study as 0.83 (good)).

VALIDITY:

- Construct validity: correlates positive with maternal gratification (r = 0.56) and mental functioning (r = 0.39) and inversely correlated with depression (r = −0.21).

REFERENCES:

Barkin, J. L., Wisner, K. L., Bromberger, J. T., Beach, S. R., Terry, M. A., & Wisniewski, S. R. (2010). Development of the Barkin Index of Maternal Functioning. *Journal of Women's Health*, *19*(12), 2239–2246. http://doi.org/10.1089/jwh.2009.1893

Barkin, J. L., McKeever, A., Lian, B., & Wisniewski, S. R. (2017). Correlates of Postpartum Maternal Functioning in a Low-Income Obstetric Population. *J Am Psychiatr Nurses Assoc, 23*(2), 149-158. doi:10.1177/1078390317696783

Physician History-Taking Form (PHTF)

Developed by the team.

DESCRIPTION OF MEASURE: Medical and psychiatric history of patient and family. Includes pregnancy and birth information, medications, comorbid conditions and treatment of conditions.

SCORING: None

Quick Sheets for Physicians to Interpret Scores of Measures on Redcap

**Scale Name**	**Construct Measured**	**Range of Score**	**Interpretation / Cut-off of Score**
Edinburgh Postnatal Depression Scale (EPDS)	Maternal depression	0–30	Higher score = greater depressive symptoms<br><br>Total score of 13 or higher are in clinical range, at risk for depressive disorder<br><br>Item 10 *(The thought of harming myself has occurred to me)*: any score greater than 0 should be evaluated for safety of the mother and baby
Generalized Anxiety Disorder 7-Item (GAD-7) Scale	Level of maternal anxiety	0–21	Higher scores = greater symptoms of anxiety<br><br>Cut-off of 5 for mild anxiety symptoms<br>Cut-off of 10 for moderate anxiety symptoms<br>Cut-off of 15 for severe anxiety symptoms
Antenatal Risk Questionnaire (ANRQ)	Maternal prenatal psychosocial risk (for developing postnatal depression)	7–60	Higher score = greater risk<br><br>Total score above **23** indicates presence of significant risk factors
Postnatal Risk Questionnaire (PNRQ)	Maternal postpartum psychosocial risk	10–77	Higher scores = greater risk<br><br>Total score above **2**4indicates presence of significant risk factors
Cambridge Worry Scale	Maternal worry	0–80	Higher scores = more distress<br><br>No cut-off score at this time
Relationship Questionnaire (RQ)	Adult–peer relationship style	1–7 for each relationship style	Higher score = participant feels style describes them<br><br>Question 1: participant selects style that is most like them (secure, fearful, preoccupied, dismissing)<br><br>Question 2: participant rates how much each attachment style is like them (7 being most like them)

Scale Name	Construct Measured	Range of Score	Interpretation / Cut-off of Score
Adverse childhood experience (ACE) questionnaire	Adverse childhood experiences	0–10	Higher scores = greater adverse childhood experience exposure (1 point per experience)<br><br>No cut-off
Postpartum bonding questionnaire (PBQ)	Mother–infant bonding	Factor 1: 0 –60<br>Factor 2: 0–35<br>Factor 3: 0–20<br>Factor 4: 0–10<br>Total score: 0–125	Higher score = problem with mother–infant bonding<br><br>Factor 1 cut off: 11 = high (below 11 is normal)<br>General factor, identifies if there is a problem with the mother–infant relationship<br><br>Factor 2 cut off: 16 = high (below 16 is normal)<br>Identifies severe infant–mother relationship disorders (e.g., bonding disorders, threatened rejection, established rejection, pathological anger)<br><br>Factor 3 cut off: 9 = high (less than 9 is normal)<br>Infant-focused anxiety<br><br>Factor 4 cut off: 2 = high (less than 2 is normal)<br>Identification of dangerous mothers<br><br>Total score:<br>Cut off: 26 = above 26 suggests some kind of disorder<br>Cut off for 40 = cut-off for severe disorders
Pregnancy Risk Assessment Monitoring System Questionnaire (PRAMS)	Presence of risk factors in the 12 months leading up to birth	N/A	None
Barkin Index of Maternal Functioning (BIMF)	Measuring of functioning of mothers during the first year of their infant's life	0–120	Higher score = greater level of maternal functioning<br><br>No clinical cut-off (optimal functioning is 120)
